# Differential expression, molecular cloning, and characterization of porcine beta defensin 114

**DOI:** 10.1186/s40104-019-0367-0

**Published:** 2019-07-19

**Authors:** Guoqi Su, Kunhong Xie, Daiwen Chen, Bing Yu, Zhiqing Huang, Yuheng Luo, Xiangbing Mao, Ping Zheng, Jie Yu, Junqiu Luo, Jun He

**Affiliations:** 1 Animal Nutrition Institute, Sichuan Agricultural University, Chengdu, 611130 People’s Republic of China; 2 Key Laboratory for Animal Disease-Resistance Nutrition of China Ministry of Education, Sichuan Agricultural University, Chengdu, 625014 People’s Republic of China

**Keywords:** Antimicrobial activity, DLY, *E. coli*, Porcine beta defensin 114, Tibetan

## Abstract

**Background:**

β-defensins have attracted considerable research interest because of their roles in protecting hosts from various pathogens. This study was conducted to investigate the expression profiles of the porcine β-defensin 114 (*PBD114*) in different breeds and in response to infections. Moreover, the function of PBD114 protein was partially investigated.

**Methods:**

Six Tibetan pigs (TP) and six DLY (Duroc×Landrace×Yorkshire) pigs were slaughtered to explore the expression profiles of *PBD114* in different breeds and tissues. For infection models, sixteen DLY pigs were divided into two groups and challenged either with sterile saline or *E. coli* K88. The recombinant protein PBD114 (rPBD114) was obtained by using a heterologous expression system in *E. coli*.

**Results:**

*PBD114* gene was highly expressed in tissues such as the intestine, liver, spleen, and thymus. Interestingly, the expression level of *PBD114* gene was higher in the TP pigs than in the DLY pigs (*P* < 0.05), and was significantly elevated upon *E. coli* K88 challenge (*P* < 0.05). The nucleotide sequences of *PBD114* from Tibetan and DLY pigs was identical, and both showed a 210-bp open reading frame encoding a 69-amino acid mature peptide. To explaore the function of PBD114 protein, *PBD114* gene was successfully expressed in *E. coli* Origami B (DE3) and the molecular weight of the rPBD114 was estimated by SDS-PAGE to be 25 kDa. The rPBD114 was purified and mass spectrometry verified the protein as PBD114. Importantly, rPBD114 showed antimicrobial activities against *E. coli* DH5α and *E. coli* K88, and the minimal inhibitory concentrations (MICs) were 64 and 128 μg/mL, respectively. Hemolytic and cytotoxicity assays showed that rPBD114 did not affect cell viability under physiological concentrations.

**Conclusions:**

*PBD114* is an infection response gene that is differentially-expressed between different porcine breeds and tissues. The antimicrobial activity of PBD114 protein, against pathogens such as the *E. coli* K88, suggested that it may serve as a candidate for the substitution of conventionally used antibiotics.

**Electronic supplementary material:**

The online version of this article (10.1186/s40104-019-0367-0) contains supplementary material, which is available to authorized users.

## Introduction

The availability of antibiotics for treating bacterial infections has significantly improved the health of animals and humans. In addition, sub-therapeutic levels of antibiotics have been added to pig feed as growth promoters in many countries [1]. However, liberal antibiotic use combined with the ability of microorganisms to develop antibiotic resistance has led to multi-drug resistant, which has fuelled a potential global public health crisis [2]. To reduce the emergence of antimicrobial resistance, approaches to reduce the utilization of antibiotics in animal production are needed.

Innate immunity is the first line of host defense against invading pathogens such as bacteria, virus, and fungus. Previous studies have indicated that one of the primary innate immune responses are the secretion of antimicrobial peptides (AMPs) [3, 4]. To date, thousands of antimicrobial peptides (AMPs) have already been identified in vertebrates, invertebrates, plants and fungi (http://aps.unmc.edu/AP/main.php). AMPs are classified based on structural and sequence homology, with host defence peptides (HDPs) comprising one of the major subclasses of the family of AMPs [5]. HDPs are a family of low molecular weight peptides secreted by organisms, and can protect hosts from a broad range of pathogens including bacteria, virus and fungus [3–5]. Upon microbial invasion, mature active HDPs are released quickly by proteolytic processing from precursor peptides either in cytoplasmic granules of most mammalian neutrophils and macrophages or in epithelial cells of the respiratory, gastroenteric, and urogenital tracts [6, 7]. HDPs disrupt the bacterial membrane by forming non-specifc electrostatic interactions with the membrane lipid components [8]. So bacteria are less able to develop resistance to HDPs than to traditional antibiotics. Therefore, the administration of HDPs is a potentially novel therapeutic strategy for inflammatory and infectious diseases of the gastrointestinal tract [7, 9], and may present a promising alternative to the traditional antibiotic feed additives used in the livestock industry.

The first identified porcine beta defensin was porcine beta defensin 1 (PBD1), which can be detected throughout the respiratory and digestive tracts [6]. Further studies found that PBD1 displayed strong antimicrobial activity against *Bordetella pertussis*, *Staphylococcus aureus* and multi-resistant *E. coli* [10–12]. In addition, previously reported that porcine beta defensin (PBD2) was expressed in the intestine, and mature PBD2 synthetic peptide exhibited high antimicrobial activity against a broad range of pathogenic bacteria, but only limited hemolytic activity against porcine red blood cells [1, 12]. A recent study reported that there were at least 29 different β-defensins expressed in porcine tissues. The average nucleotide sequence identity from the 27 pairs of orthologous β-defensins between humans and pigs was 84.38%, and this probably indicated that β-defensins have multiple biological functions [13]. Although the porcine beta defensins may function as an important regulator of host defense against exogenous pathogens, the expression profile and biological functions of PBD114 are still unclear. The present study was conducted to explore the expression profiles of the *PBD114* in different breeds and in response to infections. Moreover, the function of PBD114 protein has also been investigated.

## Materials and methods

### Strains and vectors

*E. coli* DH5α and *E. coli* Origami B (DE3) strains were purchased from TIANGEN (Beijing, China). *E. coli* K88 was kindly provided by Professor Lianqiang Che, Institute of Animal Nutrition, Sichuan Agricultural University. *Salmonella typhimurium (S. typhimurium)* ATCC14028, *Staphylococcus aureus* (*S. aureus*) CICC10384, *Streptococcus*, *Pichia pastoris* X33 and *Bacillus subtilis* (*B. subtilis*) were kindly provided by Professor Qigui Yan, College of Animal Science and Technology, Sichuan Agricultural University. The plasmids pMD19-T Simple and pET32a (+) were purchased from Takara (Dalian, China) and Merck KGaA (Darmstadt, Germany) respectively.

### Animals

Six 60 days old TP (6.63 ± 0.85 kg) and six 60 days old DLY (21.72 ± 0.36 kg) pigs were purchased from the animal husbandry science research institute, Ganzi Tibetan Autonomous Prefecture in Sichuan Province. All pigs were immediately euthanized to collect liver, spleen, kidney, lung, thymus, duodenum, jejunum, ileum, cecum and colon, then collected tissues were immediately snap-frozen in liquid nitrogen for quantitative real-time PCR.

Sixteen 7 days old DLY pigs (3.14 ± 0.07 kg) were purchased from the Sichuan Giastar Group. All pigs were randomly divided into two groups: CON and K88. Pigs of K88 group and CON group were given a gavage of 80 mL 1 × 10^9^ CFU/mL *E. coli* K88 and the same volume normal saline respectively. Diarrhoea scores were recorded every 4 h for a total 24 h after challenge. Artificial milk (Table 1) was fed every 3 h during the study period. After the experiment, all pigs were euthanized to collect duodenum, jejunum, ileum, cecum and colon, and then immediately snap-frozen in liquid nitrogen for quantitative real-time PCR.

**Table 1:** Composition and nutrient content of artificial milk (87.5% DM basis)

Items	Control
Ingredients, %	
Whole milk powder, 24% CP	58.00
Whey protein concentrate, 34% CP	25.00
Casein	2.00
Coconut oil	12.40
CaH_2_PO_4,_ 22%P	0.10
Choline chloride, 50%	0.10
Vitamin mixture^a^	0.10
Mineral mixture^b^	0.50
*L*-Alanine, 95%	0.85
*L*-Leu, 95%	─
*L*-Arg, 98.5%	0.06
*DL*-Met, 98.5%	0.06
*L*-Lys.HCl, 78.5%	0.75
*L*-Thr, 98%	0.03
*L*-Trp, 98%	0.05
Nutrient content^c^	
Crude protein, %	26.13
Digestible energy, MJ/kg	19.21
Calcium, %	1.02
Total phosphorus, %	0.81
Available phosphorus, %	0.67
Total isoleucine, %	1.24
Total leucine, %	2.40
Total valine, %	1.36
Total lysine, %	2.19
Total methionine, %	0.79
Total arginine, %	0.77

*CP* crude protein

^a^Vitamin premix provided per kg powder diet: vitamin A, 0.94 mg; vitamin D_3_, 0.01 mg; vitamin E, 20 mg; vitamin K_3_, 1 mg; vitamin B_12_, 0.04 mg; riboflavin, 5 mg; niacin, 20 mg; pantothenic acid, 15 mg; folic acid, 1.5 mg; thiamin, 1.5 mg; pyridoxine, 2 mg; biotin, 0.1 mg

^b^Mineral premix provided per kg powder diet: Zn, 90 mg; Mn, 4.0 mg; Fe, 90 mg; Cu, 6.0 mg; I, 0.2 mg; Se, 0.3 mg

^c^All nutrient contents, except digestible energy, were measured

### Cell culture

Intestinal porcine epithelial cells (IPEC-J2) were cultured in 75 cm^2^ cell culture flask in DMEM-F12 with 10% FBS, 100 U/mL penicillin, and 100 μg/mL streptomycin. 1 × 10^5^ cells/well were seeded in 12-well plates and grown to ~ 80% confluence at 37 °C in a CO_2_ incubator (5% *v*/*v*), then cultured for 12 h in culture medium without FBS and penicillin-streptomycin. Cells were challenged with 1 × 10^5/6/7^ CFU/well *E. coli* K88 for 1.5 h, control cells were not challenged. Total cellular RNA was collected using RNAiso Plus (Takara, Dalian, China).

### Total RNA isolation and PCR

Total RNA was isolated from pigs using RNAiso Plus (Takara, Dalian, China) according to the manufacturer’s instructions. The quantity and quality of the isolated RNA were determined by absorbance at 260 and 280 nm [14]. And then cDNA was synthesized using a Reverse Transcriptase kit (Takara, Dalian, China). Subsequently, *PBD114* cDNA was amplified by PCR using the following specific primers which were designed online: forward primer F (5'-CCGGAATTCACCTTGGTGGATCCTGAACG-3') and reverse primer R (5'-TTGCGGCCGCAGATAAATCTTCTTCAAACGC-3') supplemented with *Eco*R I/*Not* I restriction sites. The PCR reaction was performed according the manufacturers’ instructions of 2 × PCR Solution Premix Taq™ (Takara, Japan). The amplified PCR products were collected and purified by a DNA Fragment Purification Kit (Takara, Dalian, China). Then the amplified products of *PBD114* by PCR were directly cloned into the pMD19-T Simple Vector, positive clones were identified by colony PCR and sequenced by Sangon Biotech (Shanghai, China). Sequence alignment of *PBD114* from TP and DLY were performed by DNAMAN 8.0, and phylogenetic tree of beta defensin 114 among human, *Sus scrofa*, *Bos indicus* and *Pan troglodytes* was analyzed with DNAMAN 8.0. Quantitative real-time PCR was performed on the ABI PRISM 7500 Fast Sequence Detection System for ninety-six well plates (Applied Biosystems) using the SYBR green 2× RT-PCR mix (Takara, Dalian, China). The primers of *β-actin*, *TNFα* and *PBD114* are shown in Table 2, and the gene *β-actin* was used as a housekeeping gene. The relative gene expressions compared with the housekeeping gene *β-actin* were calculated by 2^-CT^ [15].

**Table 2:** Primers sequences used for quantitative RT-PCR

Gene	Accession No.	Primer sequences (5′→3′)	Product length, bp
*β-actin*	XM_003124280.5	TGGAACGGTGAAGGTGACAGC	177
		GCTTTTGGGAAGGCAGGGACT	
*TNFα*	NM_214022.1	GCATCGCCGTCTCCTACCAG	173
		GGGCAGGTTGATCTCGGCAC	
*PBD114*	NM_001129973.1	TTGGTGGATCCTGAACGATGCT	130
		CTTCTTCAAACGCCCTCTGAATGC	

### Preparation of rPBD114

Gene *PBD114* (without signal peptide) was synthesized by TsingKe (Beijng, China) and inserted into expression vector pET32a(+) to construct recombinant expression vector pET32a(+)-PBD114. The construction of the engineered strain *E. coli* Origami B (DE3)-pET32a(+)-PBD114, induction of recombinant engineered strain and SDS-PAGE were performed according previous study [16], the engineered strain *E. coli* Origami B (DE3)-pET32a(+) was used as negative control. Identification was carried out through mass spectrometry (MALDI-TOF/TOF) by Beijing Genomics Institute. Purification of recombinant PBD114 protein (rPBD114) referred to our previous study [16]. Briefly, the supernatant with crude rPBD114 was applied to Ni^2+^-IDA column (Sangon Biotech, Shanghai, China) and purified according specification. Ten resin volumes of Binding Buffer (50 mmol/L NaH_2_PO_4_, 300 mmol/L NaCl, pH 8.0) were added to wash way the impure protein, and then five resin volumes of Elution Buffer (50 mmol/L NaH_2_PO_4_, 300 mmol/L NaCl, 150 mmol/L imidazole, pH 8.0) were added to eluted the rPBD114 from the column. The protein concentration was determined by the BCA assay (Beyotime, Shanghai, China). The purifed rPBD114 was electrophoresed on a 12% SDS-PAGE gel. The rest was stored at − 80 °C to analyze biological activities.

### Structure homology-modelling and physical and chemical parameters of PBD114

The automated protein structure homology-modelling of PBD114 was performed by SWISS-MODEL online (https://swissmodel.expasy.org/) to predict the structure of PBD114. The computation of various physical and chemical parameters for PBD114 carried out by ExPASy online (https://web.expasy.org/protparam/) to predict theoretical pI, net charged residues and aliphatic index of PBD114.

### Minimal inhibitory concentration

The minimal inhibitory concentration (MIC) for antimicrobial activity analysis of purified rPBD114 was measured by the microtiter broth dilution method [17]. *E. coli* DH5α, pathogenic *E. coli* K88+, *S. typhimurium*, *S. aureus*, *B. subtilis* were grown to 0.4 OD_600_ at 37 °C in LB, *Streptococcus* was grown to 0.4 OD_600_ at 37 °C in THY (Todd-Hewitt+yeast extract), *Pichia pastoris* X_33_ was grown to 0.4 OD_600_ at 37 °C in YPD. The target cell culture was diluted to 1 × 10^5^ CFU/mL with same media respectively. A total of 100 μL of rPBD114 and 100 μL of cell suspension were added into each well. The activity of rPBD114 was tested over a concentration range of 256, 128, 64, 32, 16, 8, 4, 2, 1, 0.5 and 0.25 μg/mL, and all assays were tested in triplicated. Bacterial plates were incubated at 37 °C for 16 h, fungal plate was incubated at 30 °C for 24 h, and the absorption of cell culture was recorded at 600 nm. MIC was defined as the lowest concentration of peptide at which there was no change in optical density.

### Hemolytic activity assay

The hemolytic activity of rPBD114 was measured spectrophotometrically using a hemoglobin release assay [18]. Fresh pig blood was collected to prepare erythrocytes by centrifugation at 1500 r/min for 10 min at room temperature. The erythrocytes were gently washed three times with PBS (pH 7.2) and resuspended to 4% (*v*/*v*) with PBS (pH 7.2). 4% erythrocytes were incubated with different concentrations (0.5~256 μg/mL, double gradient) of rPBD114 for 1 h at 37 °C, and then centrifuged at 1000 r/min for 5 min. Three mL supernatant were then added to a 2-cm quartz cuvette, and the absorbance was measured at 414 nm with UV-1100 spectrophotometer (ShangHai, China). No hemolysis and 100% hemolysis were determined in PBS and Triton X-100, respectively.

### Cytotoxicity assay

The cytotoxicity of rBPD114 was measured according to previously a study [19]. Briefly, IPEC-J2 cells were cultured in DMEM-F12 with 10% FBS, 100 U/mL penicillin, and 100 μg/mL streptomycin for 48 h and then resuspended to 10^5^ cells/mL in FBS free DMEM-F12 media. A volume of 100 μL of cells was aliquoted into sterile flat-bottomed 96-well plates (Corning, USA). Final concentration of 0, 4, 16, 64 and 256 μg/mL rPBD114 were added to the cells and incubated at 37 °C/5% CO_2_ for 24 h. Cell viability was evaluated with the CCK-8 assay (Beyotime, Shanghai, China) according to the manufacturer’s instructions.

### Statistical analysis

All of the statistical analyses were performed using Graphpad Prism 7.0. Differences in *PBD114* expression among breeds were investigated using Student’s *t*-test (and nonparametric tests). Treatment differences of *PBD114* expression of IPEC-J2 and cytotoxicity were statistically analyzed by One-way ANOVA, Tukey’s test was applied post hoc. Data were expressed as the mean ± standard error of the mean. Values in the same row with different superscripts are significant (*P* < 0.05), while values with same superscripts are not significant different (*P* > 0.05).

## Results

### Expression profiles of *PBD114* gene in different pig breeds and tissues

**Fig. 1:**
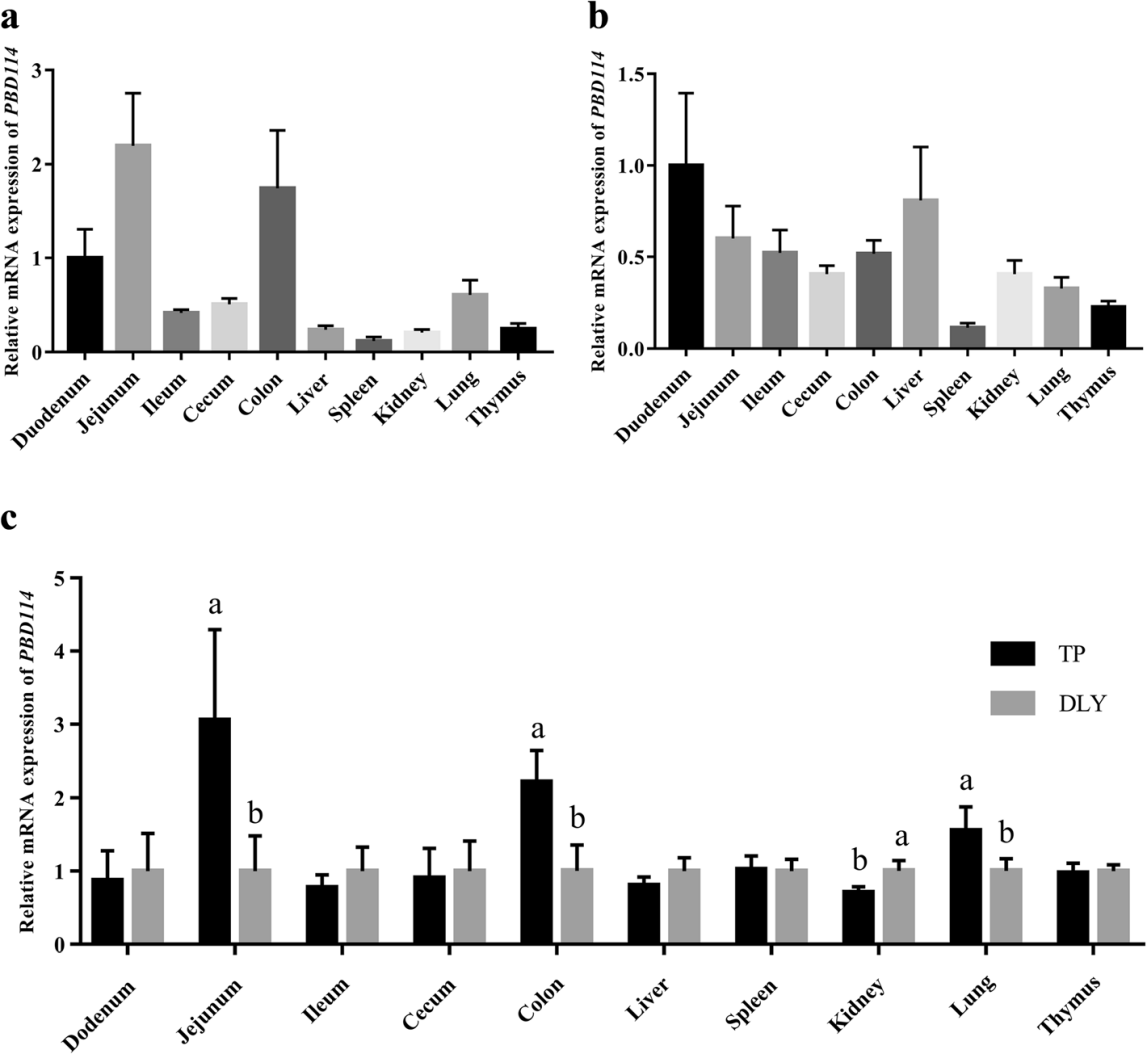
The mRNA level of *PBD114* in Tibetan and DLY pigs. **a**, the mRNA level of *PBD114* in tissues of Tibetan pigs; **b**, the mRNA level of *PBD114* in tissues of DLY pigs; **c**, comparison of the mRNA level of *PBD114* among breeds. Values are means ± SE, *n* = 6. ^a, b^ Mean in a same column with different superscripts differ significantly (*P* < 0.05)

To evaluate expression profiles of *PBD114*, mRNA expressions in various tissues among TP (Fig. 1a) and DLY (Fig. 1b) were measured. *PBD114* widely expressed in duodenum, jejunum, ileum, cecum, colon, liver, spleen, kidney, lung and thymus, while the tissue with the highest expression abundance was jejunum in TP and duodenum of DLY. The different expression levels of *PBD114* between TP pigs and DLY pigs were display in Fig. 1c. The results showed that the expression levels of *PBD114* of TP pigs in jejunum, colon and lung were higher than DLY pigs (*P* < 0.05). However, the abundance of *PBD114* of TP pigs in kidney were lower than DLY pigs (*P* < 0.05).

### Expression profiles of *PBD114* gene upon *E. coli* K88 challenge

**Fig. 2:**
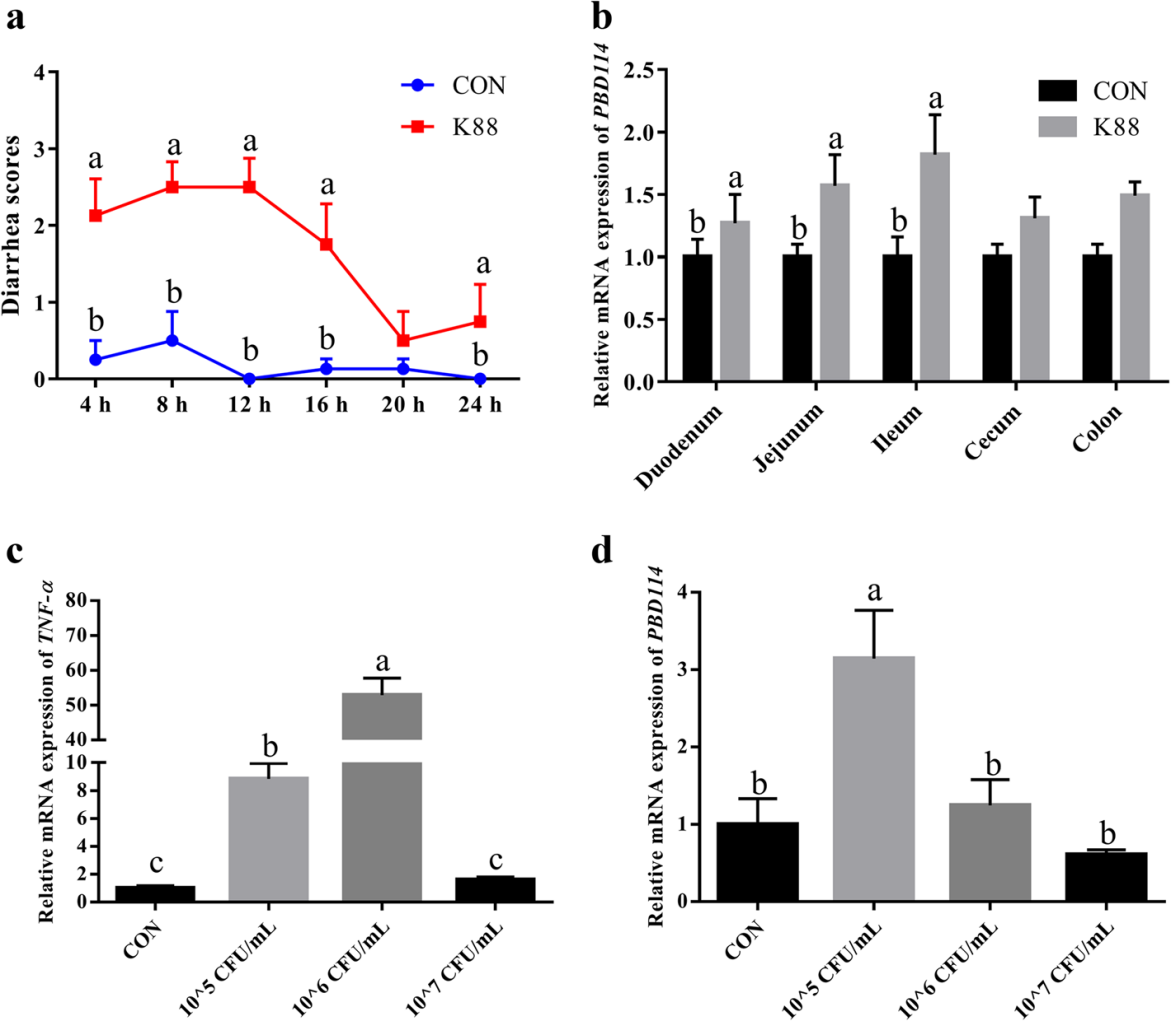
*Escherichia coli* K88 induced the expression of *PBD114* in 6 days old pigs and IPEC-J2. **a**, diarrhea scores of 7 days old piglets challenged by *E. coli* K88 or not; **b**, effects of *E. coli* K88 challenge on the mRNA level of *PBD114* in intestinal tissues; Effects of *E. coli* K88 challenge on the mRNA level of *TNFα* (**c**) and *PBD114* (**d**) in IPEC-J2. Values are means ± SE, *n* = 8. ^a, b, c^ Mean in a same column with different superscripts differ significantly (*P* < 0.05)

The effects of *E. coli* K88 on the expression of *PBD114* were displayed in Fig. 2. Diarrhea scores of K88 group were higher than CON group over 24 h except at 20 h (*P* < 0.05), and the results indicated that *E. coli* K88 challenged model was successful. *In vivo*, the expressions of *PBD114* in duodenum, jejunum and ileum were significantly induced by *E. coli* K88 (*P* < 0.05). *In vitro*, the expressions of *TNFα* in IPEC-J2 were markedly increased by 1 × 10^5^ and 1 × 10^6^ CFU/mL *E. coli* K88, and 1 × 10^5^ CFU/mL *E. coli* K88 also obviously induced the expression of *PBD114* (*P* < 0.05).

### Cloning and sequence analysis of the *PBD114* gene

**Fig. 3:**
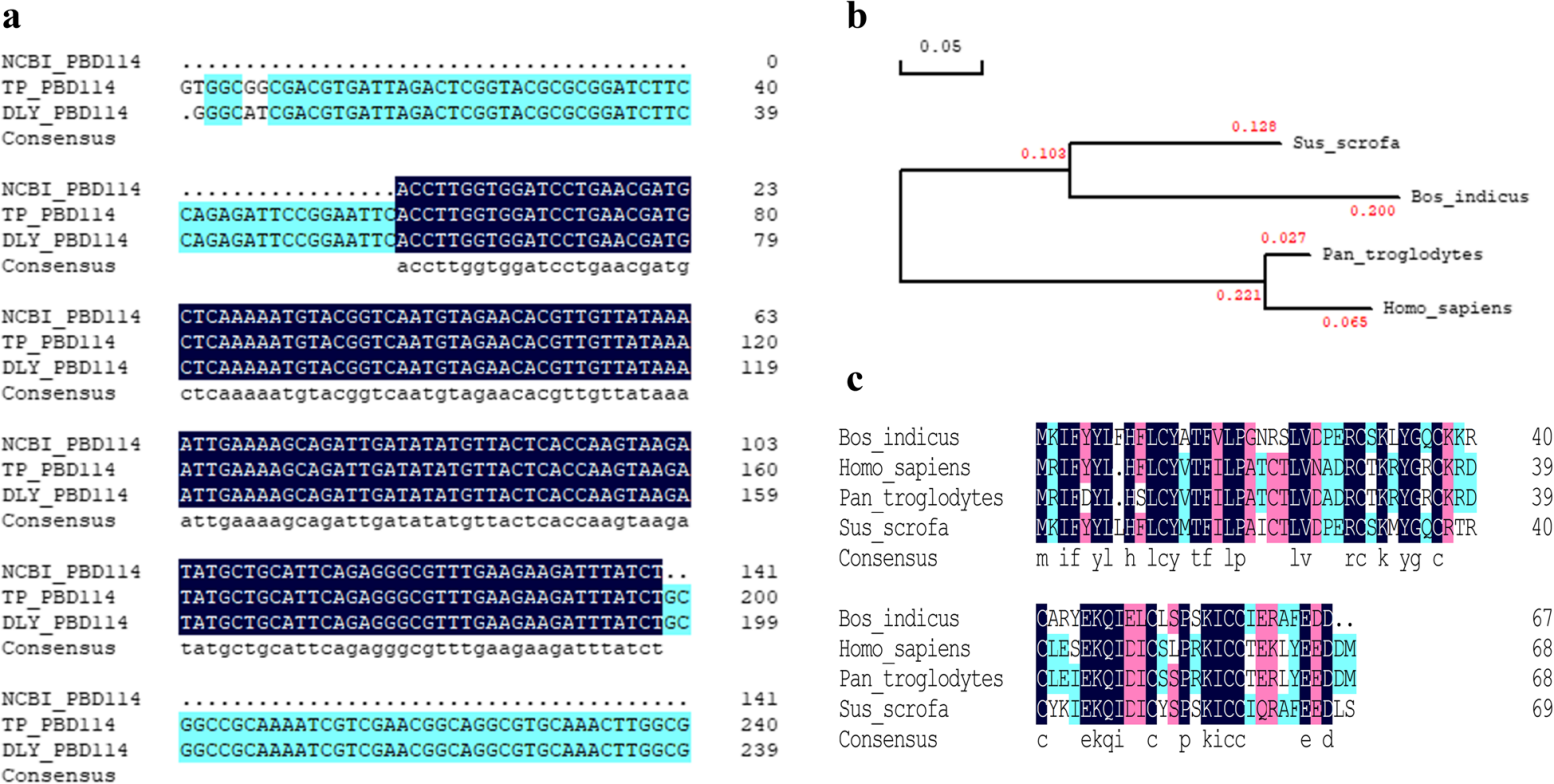
Sequence alignment of PBD114 and phylogenetic analysis of beta defensin 114. **a**, DNA sequences cloned from TP and DLY pigs were aligned by DNAMAN 8.0; **b**, amino acid sequences of beta defensin 114 in sus scorfa, *Homo sapiens*, *Pan troglodytes* and *Bos indicus* were aligned by DNAMAN 8.0; **c**, phylogenetic analysis of beta defensin 114 in sus scorfa, *Homo sapiens*, *Pan troglodytes* and *Bos indicus* were performed by DNAMAN 8.0

In order to compare the homology of *PBD114* between TP pigs and DLY pigs, the 210-bp open reading frame of PBD114 from TP pigs and DLY pigs were successfully amplified (Additional file 1: Figure S1). Then *PBD114* amplicons from TP and DLY pigs were cloned and sequenced, and the results of sequence alignment indicated that DNA sequence identity of *PBD114* between TP and DLY pigs was identical with NM_001129973.1 (Fig. 3a). Amino acid sequence identity and phylogenetic tree of beta defensin 114 among *Homo sapiens*, *Sus scrofa*, *Bos indicus* and *Pan troglodytes* was compared. Phylogenetic tree analysis results indicated that beta defensin 114 of *Sus scrofa* was close to *Homo sapiens* (Fig. 3b). Moreover, with 45.83% amino acid sequence identity of beta defensin 114 from human and *Sus scrofa* (Fig. 3c).

### Expression and purification of rPBD114

**Fig. 4:**
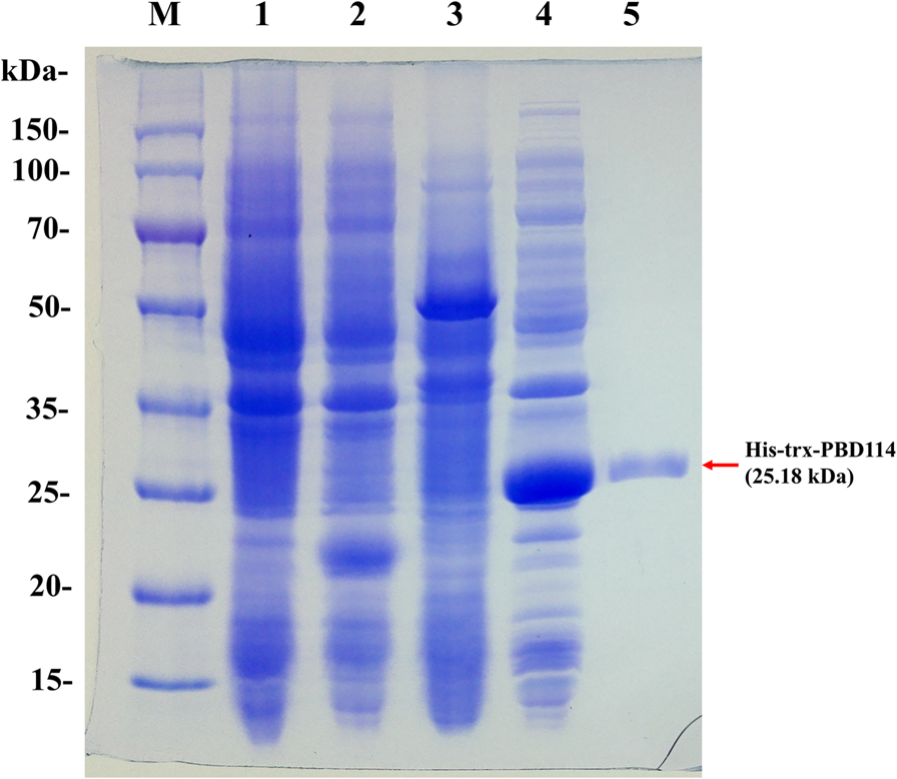
SDS-PAGE of recombinant PBD114 protein. M, 150 kDa protein marker; 1, *E. coli* Origami B (DE3)-pET32a(+) did not induce for 9 h at 28 °C; 2, *E. coli* Origami B (DE3)-pET32a(+) induced by 1 mmol/L IPTG for 9 h at 28 °C; 3, *E. coli* Origami B (DE3)-pET32a(+)-PBD114 did not induce for 9 h at 28 °C; 4, *E. coli* Origami B (DE3)-pET32a(+)-PBD114 induced for 9 h at 28 °C

**Fig. 5:**
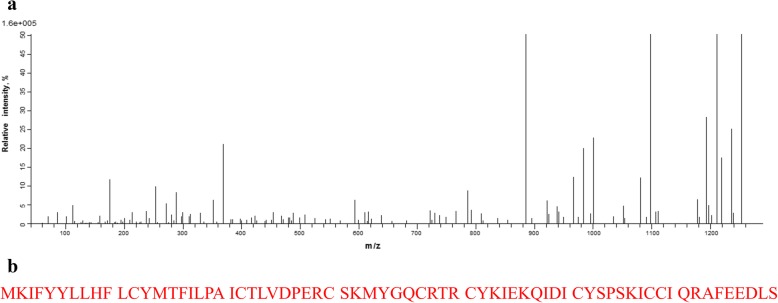
Mass spectrometry identification of rPBD114. **a**, peak figure of amino acid fragments; **b**, through searching uniprot-*Sus*-*scrofa* (68152 sequences, 25615784 residues), rPBD114 sequence had a 100% math with NP_001123445 (show in red)

The recombinant strain *E. coli* Origami B (DE3)-pET32a(+)-PBD114 and *E. coli* Origami B (DE3)-pET32a(+) were constructed, and positive colonies were identified by colony PCR (data were not showed). And then the identified positive colony was cultured and induced by 1 mmol/L isopropyl-β-d-thiogalactoside (IPTG) at 28 °C for 9 h. Then SDS-PAGE analysis showed that the target protein with the Trx tag conformed to the theoretical molecular weight (Fig. 4), of 25.18 kDa. The pellets were disrupted with sonication and dissolved in lysis buffer. The crude soluble recombinant protein was purified by Ni^2+^-IDA affinity chromatography. SDS-PAGE was performed and the purity of rPBD114 was analyzed with Image Lab (Bio-Rad). The result showed that the purity of rPBD114 was 95% and the expression level of rPBD114 was 5.0 mg/L. The target band was collected and amino acid sequence of rPBD114 was identified by mass spectrometry (MALDI-TOF/TOF) (Fig. 5a). Through searching uniprot-Sus-scrofa (68,152 sequences, 25,615,784 residues), rPBD114 sequence had a 100% math with NP_001123445 (Fig. 5b), more MS information was shown in Additional file 2 data.

### Protein structure, physical and chemical parameters of PBD114

Protein structure prediction was performed online to explore the biological function of PBD114. SWISS-MODEL analysis found that the structure of PBD114 was similar to human beta defensin 2 with 35.14% sequence identity (Additional file 1: Figure S2). The predicted structure of PBD114 indicated that PBD114 was consisted of an alpha helix fragment and a β-pleated sheet fragment (Additional file 1: Figure S2), the detail predicted results were provided in Additional file 1: Figure S3. Physical and chemical parameters of PBD114 was analyzed by ProtParam online, theoretical pI, net charged residues and aliphatic index of PBD114 was 7.46, + 1 and 66.38 respectively, and more information was shown in Additional file 3 data.

### Antibacterial activities

The antimicrobial activity of rPBD114 was explored by determining its MIC against *E. coli* DH5α, pathogenic *E. coli* K88+, *S. typhimurium*, *S. aureus*, *B. subtilis* and *Pichia pastoris* X_33_ (Table 3). The results showed that rPBD114 exhibited strong antimicrobial activity against *E. coli* DH5α and pathogenic *E. coli* K88+, The MIC value of *E. coli* DH5α and pathogenic *E. coli* K88+ were 64 and 128 μg/mL respectively. However, much higher concentration rPBD114 (more than 256 μg/mL) were needed to against *S. typhimurium*, *S. aureus*, *B. subtilis* and *Pichia pastoris* X33. The tolerance of these four organisms to rPBD114 indicate that *Pichia pastoris* X33 would be more tolerant to expression of rPBD114.

**Table 3:** MIC of rPBD114

Strain	rPBD-114, μg/mL
Gram-netative bacteria	
*E. coli* DH5α	64
Pathogenic *E. coli* K88+	128
*Salmonella typhimurium* ATCC14028	> 512
Gram-positive bacteria	
*Streptococcus*	> 512
*Staphylococcus aureus* CICC10384	> 512
*Bacillus subtilis*	> 512
Fungi	
*Pichia pastoris* X_33_	512

### Hemolytic activity and cytotoxicity assays

**Fig. 6:**
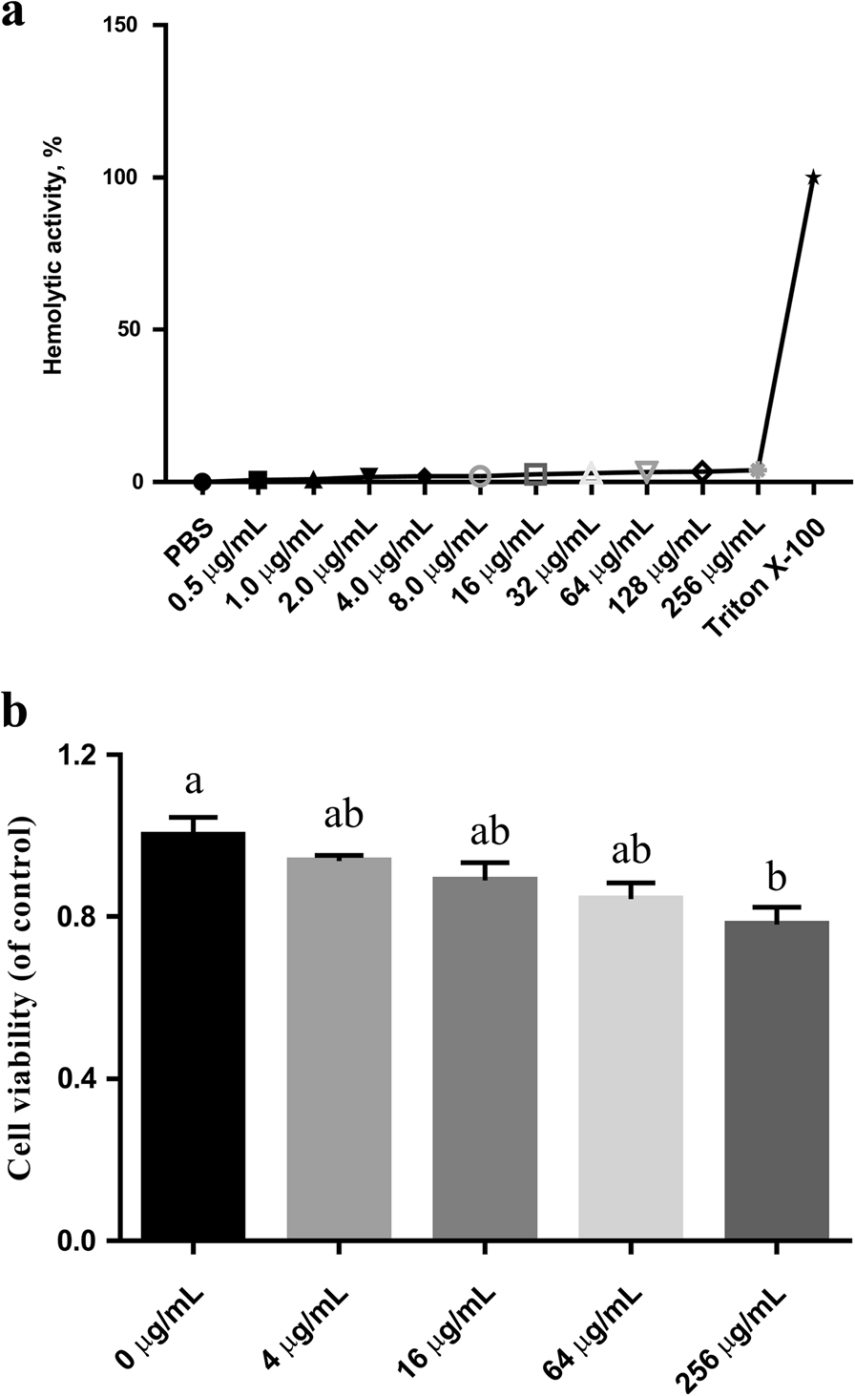
Hemolytic and cytotoxicity of recombinant PBD114 protein. **a**, hemolytic of rPBD114, porcine erythrocyte was hatched with different concentration of rPBD114 for 1 h at 37 °C and measured OD_414_; **b**, cytotoxicity of rPBD114, IPEC-J2 was co-culture with different concentration of rPBD114 for 24 h and measured by CCK8. Values are means ± SE, *n* = 6. ^a, b^ Mean in a same column with different superscripts differ significantly (*P* < 0.05)

Erythrocytes were collected from fresh porcine blood and incubated with different concentrations of rPBD114 for 1 h. Less than 3.9% hemolysis was found at all concentrations (0–256 μg/mL). The *results* showed that rPBD114 had slight hemolytic *in vitro* (Fig. 6a). IPEC-J2 was also used to examine cytotoxicity of rPBD114, cell viability was measured by CCK-8 after treating with different concentrations of rPBD114. There was little cytotoxicity (Fig. 6b) when the concentration of rPBD114 was less than 256 μg/mL. However, the cytotoxicity was significant when the concentration of rPBD114 was 256 μg/mL (*P* < 0.05). These results suggested that the safe concentration of rPBD114 should be lower than 256 μg/mL.

## Discussion

The innate and adaptive immunity are two essential elements of host defense. Innate immunity is highly conserved from fruit flies to human and is the first line of defense against invading pathogens. One mechanism of the innate immunity is the secretion of broad-spectrum antimicrobial substances, such as cathelicidins and small cationic polypeptides named defensins [20]. Previous studies reported that porcine β-defensin 1 and β-defensin 2 existed broad-spectrum antimicrobial activities and immune modulating function [20]. Human beta defensin 114 exhibited strong antimicrobial activity against *E. coli*, *C. albicans* and *S. aureus*, and anti-inflammatory function [21]. Choi et al. [13] found PBD114 by a BLAST analysis in 2012. However, the expression profile of PBD114 in various tissues of pigs and biological function of PBD114 have not been studied. Therefore, the present study was conducted to explore the expression profiles of the *PBD114* in different breeds and in response to infections. Moreover, the function of PBD114 protein has also been investigated.

Our results showed that *PBD114* widely expressed in duodenum, jejunum, ileum, cecum, colon, liver, spleen, lung, kidney and thymus, in both TP and DLY pigs. Similar expression patterns also have been demonstrated by previous study on porcine beta defensins [13]. The extensive expression of *PBD114* suggested that this endogenous peptide antibiotic may contribute to both mucosal and systemic host defenses in pigs. Chinese TP is a special Chinese indigenous pig breed, which are distributed in high-altitude areas of Qinghai-Tibet Plateau which has cold climate. They are raised in pollution-free, purely natural alpine and cold mountainous areas all year round. And their immunity and disease resistance are stronger than Duroc pigs [22, 23]. The higher level mRNA of *PBD114* was detected in intestine of TP and DLY pigs, furthermore, the results of comparison among breeds showed that the abundance of *PBD114* mRNA of TP pigs in jejunum, colon and lung were higher than DLY pigs. As well, defensins are a family of endogenous cationic antimicrobial peptides that play an important role in the innate immune system of mammals and provide protection against bacterial infections in the intestine [24, 25], Hence, the high expression of *PBD114* in TP pigs may have implications for the contribution of *PBD114* on high disease resistance of TP, and which was the same as PBD1, 2 and 3 [22, 26].

Due to the impact of thousands of years of artificial selection for survival of these breeds of different signatures of selection in TP and domestic pigs [23], we chose to investigate whether there were some variants in the *PBD114* gene. So the DNA sequence of *PBD114* was cloned from TP and DLY pigs respectively. Agarose gel electrophoresis of cloning PCR showed that the size of *PBD114* from TP and DLY pigs was same to theoretical value (210 bp). Further DNA sequence alignment of *PBD114* between TP and DLY pigs indicated that the identity was 100%, and exactly consistent with NM_001129973.1 in NCBI. The sequence of gene *PBD114* did not mutated during thousands evolutionary history, and this hinted that *PBD114* was very important to survive and breed of pigs. Moreover, previous study reported that human defensin 114 exhibited a broad spectrum of antimicrobial activity with *Escherichia coli*, *Staphylococcus aureus* and *Candida albicans* [27]. Amino acid sequence alignment of beta defensin 114 between pig and human showed that the identity was 45.83%, and phylogenetic analysis was performed with amino acid of *Sus scrofa*, *Homo sapiens*, *Pan troglodytes* and *Bos indicus*, and showed that PBD114 was closely related to DEFB114. These results suggested that PBD114 may possess antimicrobial activity like DEFB114.

To explore whether *PBD114* take part in resistance to pathogens, we carried out experiments *in vitro* and *in vivo*. The results showed that *E. coli* K88 significantly induced the expression of *PBD114* *in vitro* and *in vivo*. On the one hand, the results indicated that *PBD114* was an inducible defensin. According previous studies, *PBD114* was not only induced by *E. coli* K88, but also could be promoted by nutrients and probiotics [27, 28]. On the other hand, *PBD114* may play an important role in killing *E. coli* K88 or immune modulating function to alleviate the damage of *E. coli* K88. Because previous study reported that DEFB114 (a human homologous protein of PBD114) not only exhibited antimicrobial activity but also could inhibit RAW264.7 release TNFα after stimulation with LPS [26]. In addition, PBD2 protected intestinal health via modulating of TJ proteins in intestine and inhibiting the production of inflammatory mediators [29].

To explore the antimicrobial activity of PBD114, PBD114 was expressed in an *Escherichia coli* expression system. Because *Escherichia coli* expression system technology was mature and simple, still the first choice as host for AMPs production [16, 30]. In addition, PBD114 possessed three disulfide bonds, so we selected pET32a(+) and *E. coli* Origami B (DE3) to construct recombinant expression bacteria. pET32a(+) vector possessed thioredoxin tag which can increase the activity and amount of target protein present in the soluble fraction. *E. coli* Origami B (DE3) with glutathione reductase (gor) and/or thioredoxin reductase (trxB) mutations enhance the formation of disulfide bonds in the *E. coli* cytoplasm [31]. We successfully constructed recombinant bacteria, *E. coli* Origami B (DE3)-pET32a (+)-PBD114 and expressed the rPBD114 protein. rPBD114 was identified by mass spectrometry and the sequence coverage 100% was identical to NP_001123445. Physical and chemical parameters showed that rPBD114 possessed positive net charge (+ 1), aliphatic index (66.38) and grand average of hydropathicity (− 0.530), and suggested that PBD114 protein has the biochemical properties of antimicrobial peptides [32]. As we all know, protein structure determines protein function. To learn more about the function of PBD114 protein, we predicted the protein structure of PBD114 protein on SWISS-MODEL and the results showed that the structure of PBD114 protein was similar with human defensin 2. Human beta defensin 2 is produced by a number of epithelial cells and exhibits potent antimicrobial activity against Gram-negative bacteria and Candida, but not Gram-positive *Staphylococcus aureus* [33]. Similarly, MIC of rPBD114 protein was carried out and rPBD114 protein exhibited antimicrobial activity against *E. coli* but not Gram-positive bacteria and fungus. However, a great number of studies have shown that defensins have a wide range of antimicrobial activities [34–37]. The antimicrobial activity of rPBD114 protein in our study suggested that *Pichia pastoris* X_33_ would be more tolerant to expression of rPBD114. The methylotrophic yeast *Pichia pastoris* can grow to extremely high cell densities, enabling efficient protein production and secretion [38, 39]. The most attractive feature of this system is that the recombinant protein could be exactly folded, fully decorated and easily purified [40]. However, the yeast may have been more resistant to the defensins if it were not properly processed. Therefore, further study in *Pichia pastoris* was needed. In addition, hemolytic and cytotoxicity of rPBD114 protein indicated that rPBD114 was safe to mammals. These results suggested that rPBD114 may serve as a candidate for the replacement of conventionally used antibiotics. However, further researches are needed to investigate such as the structural and functional analysis, immunogenicity, and biologic function of rPBD114 protein *in vitro* and *in vivo*.

## Conclusions

*PBD114* is an infection response gene that is differentially-expressed between different porcine breeds and tissues. The antimicrobial activity of PBD114 protein, against pathogens such as the *E. coli* K88, few hemolytic activity and cytotoxicity suggested that it may serve as a candidate for the substitution for conventionally used antibiotics.

## Additional files

Additional file 1: PBD114 cloning PCR and predictive spatial structure of rPBD114. (DOCX 840 kb)

Additional file 2:  Detail information of MS. (XLSX 9 kb)

Additional file 3:  Detail information of physicochemical. (DOCX 19 kb)

## Data Availability

The datasets during and/or analyzed during the current study are available from the corresponding authors on reasonable request.

## Abbreviations

AMPs: Antimicrobial peptides
DEFB114: Human beta defensin 114
DLY: Duroc×Landrance×Yorkshire
IPTG: Isopropyl-β-d-thiogalactoside
LB: Lysogeny broth
LPS: Lipopolysaccharide
MALDI-TOF/TOF: Matrix-assisted laser desorption/ionization time of flight mass spectrometry
MIC: Minimal inhibitory concentration
PBD114: Porcine beta defensin 114
rPBD114: Recombinant porcine beta defensin 114
SDS-PAGE: Sodium dodecyl sulfate polyacrylamide gel electrophoresis
THY: Todd-Hewitt+yeast extract
TJ: Tight junction
TP: Tibetan pig
YPD: Yeast Extract Peptone Dextrose Medium
