# Supplementary material for: Differential expression, molecular cloning, and characterization of porcine beta defensin 114
Source: J Anim Sci Biotechnol. 2019 Jul 19;10:60. doi: 10.1186/s40104-019-0367-0 (PMC6639935; doi:10.1186/s40104-019-0367-0)
Supplement: Supplementary file 1 — PBD114 cloning PCR and predictive spatial structure of rPBD114. (DOCX 840 kb) [file 40104_2019_367_MOESM1_ESM.docx]

**Electronic Supplementay Material of Journal of Animal Science and Biotechnology**

**Differential expression, molecular cloning, and characterization of porcine beta defensin 114**

Guoqi Su^1, 2^, Kunhong Xie^1, 2^, Daiwen Chen^1, 2^, Bing Yu^1, 2^, Zhiqing Huang^1, 2^, Yuheng Luo^1, 2^, Xiangbing Mao^1, 2^, Ping Zheng^1, 2^, Jie Yu^1, 2^, Junqiu Luo^1, 2^, Jun He^1, 2^^[[1]](#footnote-1)^

^1^Institute of Animal Nutrition, Sichuan Agricultural University, Chengdu, Sichuan 611130, People’s Republic of China

^2^Key Laboratory for Animal Disease-Resistance Nutrition of China Ministry of Education, Sichuan Agricultural University, Chengdu, Sichuan 625014, People’s Republic of China


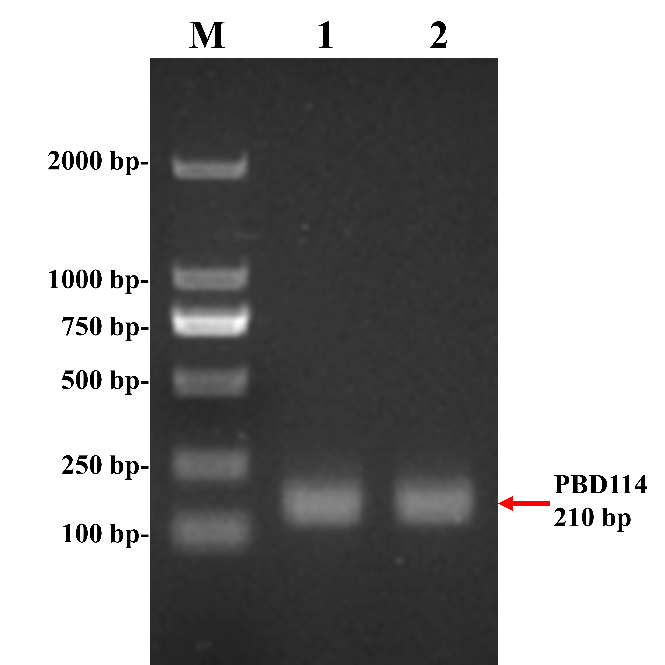


**SFig 1** Agarose gel electrophoresis of PBD114 cloning PCR

M, 2000 bp DNA marker; 1, clone PCR products of PBD114 from TP pigs; 2, clone PCR products of PBD114 from DLY pigs


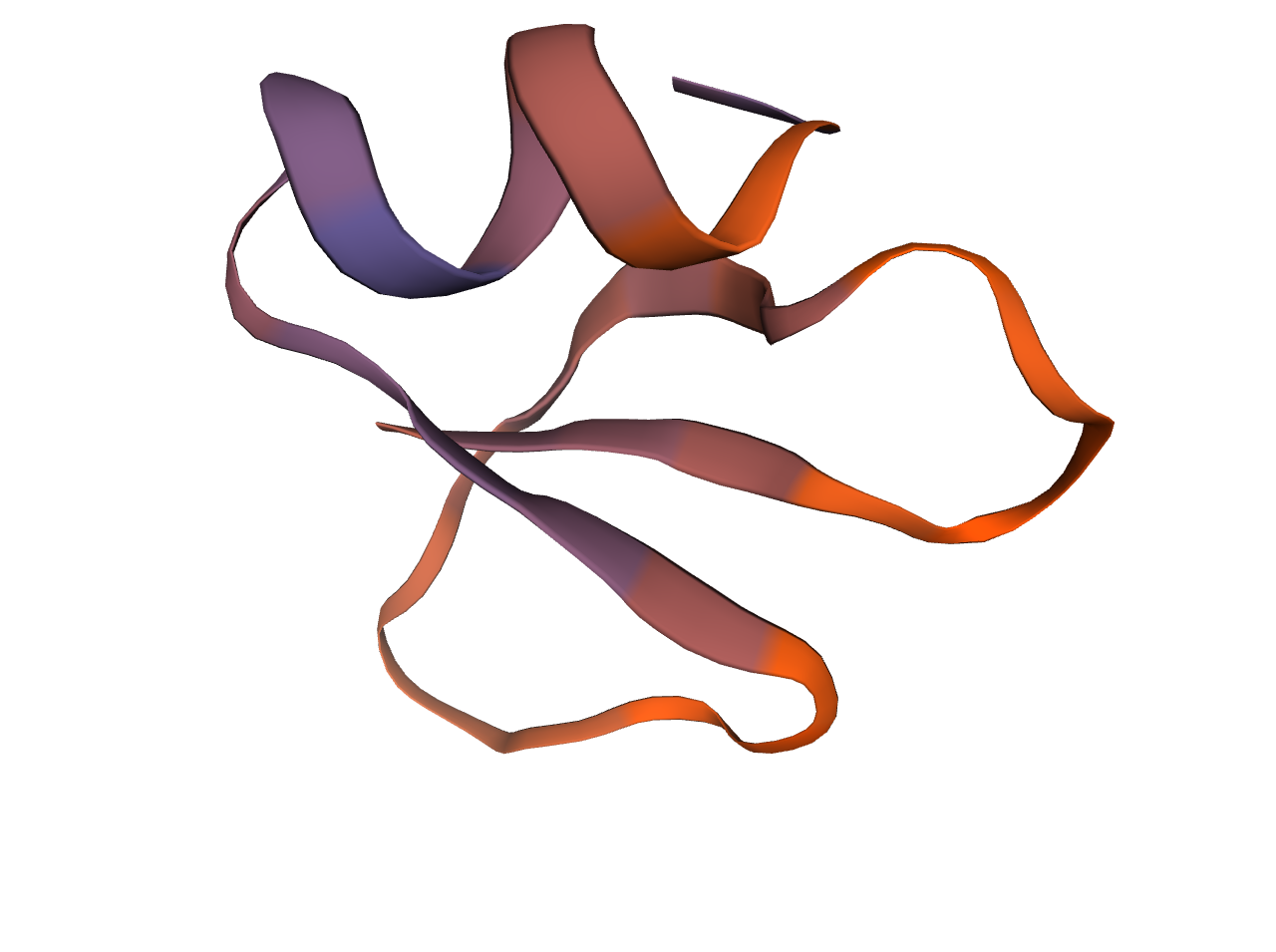


**SFig 2** Predictive spatial structure of rPBD114.


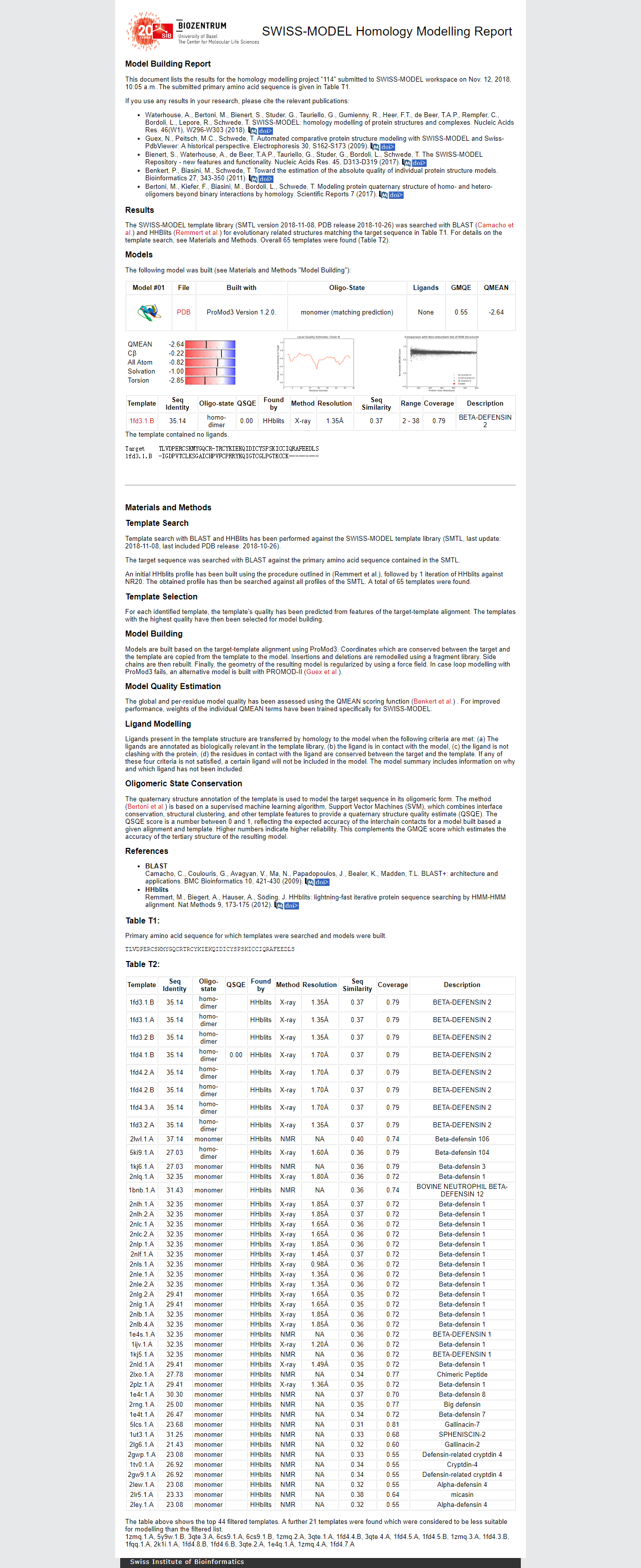


**SFig 3** Workspace of rPBD114 analyzed on SWISS-MODEL.

Second structure of rPBD114 was predicted on SWISS-MODEL (<https://swissmodel.expasy.org/>). Briefly, the amino acids of rPBD114 were input, clicked “Build Model” and run.

1. Corresponding author: Institute of Animal Nutrition, Sichuan Agricultural University, Chengdu, Sichuan 611130, People’s Republic of China; Tel: 86-835-2885065; Fax: 86-835-2885065; E-mail: hejun8067@163.com [↑](#footnote-ref-1)
