# Supplementary material for: Differential expression, molecular cloning, and characterization of porcine beta defensin 114
Source: J Anim Sci Biotechnol. 2019 Jul 19;10:60. doi: 10.1186/s40104-019-0367-0 (PMC6639935; doi:10.1186/s40104-019-0367-0)
Supplement: Supplementary file 3 — Detail information of physicochemical. (DOCX 19 kb) [file 40104_2019_367_MOESM3_ESM.docx]

**Electronic Supplementay Material of Journal of Animal Science and Biotechnology**

**Differential expression, molecular cloning, and characterization of porcine beta defensin 114**

Guoqi Su^1, 2^, Kunhong Xie^1, 2^, Daiwen Chen^1, 2^, Bing Yu^1, 2^, Zhiqing Huang^1, 2^, Yuheng Luo^1, 2^, Xiangbing Mao^1, 2^, Ping Zheng^1, 2^, Jie Yu^1, 2^, Junqiu Luo^1, 2^, Jun He^1, 2^^[[1]](#footnote-1)^

^1^Institute of Animal Nutrition, Sichuan Agricultural University, Chengdu, Sichuan 611130, People’s Republic of China

^2^Key Laboratory for Animal Disease-Resistance Nutrition of China Ministry of Education, Sichuan Agricultural University, Chengdu, Sichuan 625014, People’s Republic of China

[ProtParam](https://web.expasy.org/protparam)

[Home](https://web.expasy.org/protparam) | [**Contact**](https://web.expasy.org/contact)

**ProtParam**

**User-provided sequence:**

10 20 30 40
TLVDPERCSK MYGQCRTRCY KIEKQIDICY SPSKICCIQR AFEEDLS

[References](https://web.expasy.org/protparam/protpar-ref.html) and [documentation](https://web.expasy.org/protparam/protparam-doc.html) are available.

**Number of amino acids:** 47

**Molecular weight:** 5552.45

**Theoretical pI:** 7.46

窗体顶端

**Amino acid composition:** 
Ala (A) 1 2.1%

Arg (R) 4 8.5%

Asn (N) 0 0.0%

Asp (D) 3 6.4%

Cys (C) 6 12.8%

Gln (Q) 3 6.4%

Glu (E) 4 8.5%

Gly (G) 1 2.1%

His (H) 0 0.0%

Ile (I) 5 10.6%

Leu (L) 2 4.3%

Lys (K) 4 8.5%

Met (M) 1 2.1%

Phe (F) 1 2.1%

Pro (P) 2 4.3%

Ser (S) 4 8.5%

Thr (T) 2 4.3%

Trp (W) 0 0.0%

Tyr (Y) 3 6.4%

Val (V) 1 2.1%

Pyl (O) 0 0.0%

Sec (U) 0 0.0%

(B) 0 0.0%

(Z) 0 0.0%

(X) 0 0.0%

窗体底端

**Total number of negatively charged residues (Asp + Glu):** 7

**Total number of** **positively charged residues (Arg + Lys):** 8

**Atomic composition:**

Carbon C 236

Hydrogen H 382

Nitrogen N 66

Oxygen O 74

Sulfur S 7

**Formula:** C_236_H_382_N_66_O_74_S_7_

**Total number of atoms:** 765

**Extinction coefficients:**

This protein does not contain any Trp residues. Experience shows that

this could result in more than 10% error in the computed extinction coefficient.

Extinction coefficients are in units of M^-1^ cm^-1^, at 280 nm measured in water.

Ext. coefficient 4845

Abs 0.1% (=1 g/l) 0.873, assuming all pairs of Cys residues form cystines

Ext. coefficient 4470

Abs 0.1% (=1 g/l) 0.805, assuming all Cys residues are reduced

**Estimated half-life:**

The N-terminal of the sequence considered is T (Thr).

The estimated half-life is: 7.2 hours (mammalian reticulocytes, in vitro).

>20 hours (yeast, in vivo).

>10 hours (Escherichia coli, in vivo).

**Instability index:**

The instability index (II) is computed to be 54.11

This classifies the protein as unstable.

**Aliphatic index:** 66.38

**Grand average of hydropathicity (****GRAVY):** -0.530

1. Corresponding author: Institute of Animal Nutrition, Sichuan Agricultural University, Chengdu, Sichuan 611130, People’s Republic of China; Tel: 86-835-2885065; Fax: 86-835-2885065; E-mail: hejun8067@163.com [↑](#footnote-ref-1)
